# Dietary supplementation of β-1, 3-glucan improves the intestinal health of white shrimp (*Litopenaeus vannamei*) by modulating intestinal microbiota and inhibiting inflammatory response

**DOI:** 10.3389/fimmu.2023.1119902

**Published:** 2023-01-30

**Authors:** Kaikai Shen, Lixin Bao, Muxin Liu, Wen Lei, Qin Zhou, Jiali Ding, Peng Fang, Baoqing Hu, Chungen Wen, Vikas Kumar, Mo Peng, Gang Yang

**Affiliations:** ^1^ Department of Fisheries Science, School of Life Science, Nanchang University, Nanchang, China; ^2^ School of Animal Science and Technology, Jiangxi Agricultural University, Nanchang, China; ^3^ Aquaculture Research Institute, Department of Animal, Veterinary and Food Sciences, University of Idaho, Moscow, ID, United States

**Keywords:** *Litopenaeus vannamei*, β-1,3-glucan, intestinal microbiota, inflammatory response, immunity

## Abstract

The phenomenon of intestinal dysfunction is widely observed in white shrimp (*Litopenaeus vannamei*) culture, and β-1,3-glucan has been confirmed to be beneficial in intestinal health with a lack understanding of its underlying mechanism. Proteobacteria, Firmicutes, and Actinobacteria served as the predominant phyla inhabiting the intestine of white shrimp, whilst a significant variation in their proportion was recorded in shrimp fed with basal and β-1,3-glucan supplementation diets in this study. Dietary supplementation of β-1,3-glucan could dramatically increase the microbial diversity and affect microbial composition, concurrent with a notable reduction in the ratio of opportunistic pathogen Aeromonas, gram-negative microbes, from Gammaproteobacteria compared to the basal diet group. The benefits for microbial diversity and composition by β-1,3-glucan improved the homeostasis of intestinal microbiota through the increase of specialists’ number and inhibition of microbial competition caused by Aeromonas in ecological networks; afterward, the inhibition of Aeromonas by β-1,3-glucan diet dramatically suppressed microbial metabolism related to lipopolysaccharide biosynthesis, followed by a conspicuous decrease in the intestinal inflammatory response. The improvement of intestinal health referred to the elevation in intestinal immune and antioxidant capacity, ultimately contributing to the growth of shrimp fed β-1,3-glucan. These results suggested that β-1,3-glucan supplementation improved the intestinal health of white shrimp through the modulation of intestinal microbiota homeostasis, the suppression of intestinal inflammatory response, and the elevation of immune and antioxidant capacity, and subsequently promoted the growth of white shrimp.

## 1 Introduction

White shrimp (*Litopenaeus vannamei*), one of the most widely farmed crustaceans in the world, can be cultured in fresh seawater due to its eurysalinity (1). As one of the high density breeding species in China, white shrimp farming has been plagued by intestinal dysfunction caused by an inflammatory response, which can be induced by the infection of pathogenic bacteria from *Aeromonas*, gram-negative microbes (2). The intestinal dysfunction could ulteriorly lead to metabolic dysfunction and trigger various diseases, followed by substantial economic losses to farmers. As we know, the intestine inhabits a complex micro-ecological system, and intestinal inflammation is confirmed to be closely related to microbial dysbiosis (3). The intestine is a crucial digestion organ, while it is also an essential immunity organ for animals. And thus, finding an effective way to solve the problem of intestinal dysfunction would promote the rapid development of shrimp culture.

β-glucan is a well-known immunostimulant, which can be extracted from cell walls of fungi, algae, and natural bioactive polysaccharides and contains three different specific glycoside linkage sources, including β- (1, 3)/β- (1, 4)/β- (1, 5) (4). The β-glucan has been widely applied to the aquaculture industry due to its positive impact on the health of aquatic animals (5, 6). Numerous studies in β-1,3-glucan have confirmed its property of anti-inflammatory, immune-enhancing, and anti-oxidation in aquatic animals such as white shrimp (7–9), marine swimming crab (*Portunus trituberculatus*), hybrid striped bass (*Morone chrysops* × *M. saxatilis*) (10), and large yellow croaker (*Pseudosciaena crocea*) (11). Evidence from human hepatocytes suggested that β-1,3-glucan could attenuate inflammation and accumulation of reactive oxygen species (ROS) induced by lipopolysaccharide (12). Similarly, the results in rainbow trout (*Oncorhynchus mykiss*) (13), silver catfish (*Rhamdia quelen*) (14), and common carp (*Cyprinus carpio* L) (15) suggested that β-glucan (including β-1,3-glucan) could reduce the intestinal inflammatory response and oxidative stress induced by the challenge of *Aeromonas hydrophila* or tissue damage. Additionally, the study in Indian major carp (*Labeo rohita*) suggested that β-1,3-glucan supplementation could dramatically elevate antibody response after being challenged with the *Edwardsiella tarda* vaccine (16).

Multitudinous evidence has confirmed that a vital role of intestinal microbiota in the intestinal health of the host, and β-glucan, a complex polysaccharide, is authorized to possess various health-promoting properties through intestinal microbiota regulation (17–19). Several studies explored the effects of β-glucan on the intestinal microbiota of aquatic animals including common carp (20), tilapia (21), rainbow trout (1), and white shrimp (22), mainly focusing on microbial composition. Undoubtedly, the number and richness of species are essential elements for a microbial ecosystem, while complex interspecific interactions are crucial to maintaining intestinal microbiota homeostasis (23). And thus, β-glucan supplementation altered the composition of intestinal microbial community and would further affect interactions between different species within the microbial community. It is well accepted that the microbes perform different roles due to their ecological niches, and the critical microbes were considered essential in maintaining intestinal microbiota homeostasis (24). Since 100-fold more genes than hosts possessed by microbes could synthesize many enzymes and other products, these symbiotic microbes inhabiting the intestine could participate in host nutrition metabolism and affect intestinal health through their metabolites (25–27). Hence, the intestinal microbiota is considered as an auxiliary metabolic organ involved in the metabolic process of the host (28).

Although β-1,3-glucan displayed positive effects on intestinal health, mechanisms explaining observed immunomodulatory in white shrimp have mainly remained obscure; hence, the purpose of the current study was to explore the underlying mechanism of β-1,3-glucan improving intestinal health of white shrimp through the investigation of its effects on the intestinal microbiota, inflammatory response, immunity, and antioxidant capacity. The results from this study would provide a better understanding of the mechanism of β-1,3-glucan ameliorating the intestinal health of white shrimp and further promote the application of β-1,3-glucan in the shrimp culture.

## 2 Materials and methods

### 2.1 Experimental procedure and sample collection

Healthy juvenile freshwater-acclimated *Litopenaeus vannamei* were provided by Huachuang Ecological Agriculture Development Co., LTD (Fuzhou, China). Before the experiment, shrimp were acclimatized in a filtered, aerated freshwater cage (2.0 × 2.0 × 1.5 m) to adapt to experimental conditions for two-week. In this study, a commercial feed (Charoen Pokphand) serves as a basal diet, and its ingredients are shown in **Table 1**. After being fasted for 24 h, shrimps with an average weight of 1.21 g were randomly distributed into six cages (1.0 × 1.0 × 1.5 m) in an outdoor pond at a density of sixty per cage. The β-1,3-glucan (65% purity), extracted from *Euglena gracilis*, was provided by Yunnan Shangri-la Zeyuan Algae Industry Health Technology Co., LTD. The glucan diet adds 0.325g/kg β-1,3-glucan to the basal diet. And then, the shrimp were fed satiated with basal diet (Control group, three replicates) and glucan diet (GL group, three copies) at 06: 00, 12: 00, and 18: 00 per day for two months, respectively. Environmental conditions were monitored during the experiment: dissolved oxygen > 5.0 mg L^-1^; NH4^+^-N< 0.2 mg L^-1^; NO^2–^N< 0.1mg L^-1^.

**Table 1:** Nutritional compositions of basal diets.

Ingredients	Content (%)	Ingredients	Content (%)
Crude protein	≥ 41.0	Moisture	≤ 12.0
Crude fat	≥ 4.0	Lysine	≥ 2.1
Crude fiber	≤ 3.0	Total phosphorus	≥ 1.0
Crude ash	≤ 16.0		

At the termination of the feeding trial and following a 24 h starvation period, the total number and weight of the shrimps per cage were recorded. Five shrimps per cage were weighed and measured for condition factor (CF). After sterilization of surface skin with 70% ethanol, hemolymph samples were obtained from the ventral sinus of three shrimp. The serum was separated from hemolymph and then stored at −80°C for subsequent analysis. Additionally, midgut tissues from three shrimp per cage were stored at −80 °C. Additionally, the whole intestine containing digest from two shrimp per cage (6 samples in each group) was stored in sterile freezing tubes under −80°C.

### 2.2 16S rRNA sequencing and intestinal microbiota analysis

According to previous studies (29), the genomic DNA of microorganisms was extracted from intestinal samples and performed High-throughput sequencing after amplifying the 16S rRNA V3-V4 region using the Illumina HiSeq platform. After the quality control, a total of 1,038,520 clean reads were clustered into operational taxonomic units (OTUs) based on the Ribosomal Database (rdp_16s_v16_sp), and then performed α-diversity (Observed OTUs, Chao1, AEC, Shannon, Simpson, and invSimpson), Treemap, and Principal coordinates analysis (PCoA) based on the Bray-Curtis dissimilarity using Rstudio (30). The ecological network was constructed with a random matrix theory (RMT)-based approach (31) and then was visualized using Circos (30) and Cytoscape 3.9.0 (32). The KEGG pathway of OTUs was analyzed by PICRUSt2 software (33) and visualized by Rstudio and software STAMP (34).

### 2.3 Intestinal gene expression

Total RNA samples were extracted from midgut tissue using the Trizol reagent (Takara, Dalian, China), and cDNA was synthesized using a SMART cDNA Synthesis Kit (Clontech Laboratories, Palo Alto, CA). For quantitative real-time PCR, the specific primer pairs were shown in **Table 2**. qRT-PCR reactions were carried out in a BIO-RAD CFX96 touch q-PCR system (Applied Biosystems Inc., USA). The 2^−ΔΔCT^ method was used to compute relative gene expression levels.

**Table 2:** The primers for quantitative real-time PCR.

Primer name	Forward primers (5′ - 3′)	Reverse primers (5′ - 3′)
β-actin	GAGCAACACGGAGTTCGTTGT	CATCACCAACTGGGACGACATGGA
Crustin	GAGGGTCAAGCCTACTGCTG	ACTTATCGAGGCCAGCACAC
Penaiedin 3a	CACCCTTCGTGAGACCTTTG	AATATCCCTTTCCCACGTGAC
LZM	TGTTCCGATCTGATGTCC	GCTGTTGTAAGCCACCC
LGBP	CATGTCCAACTTCGCTTTCAGA	ATCACCGCGTGGCATCTT
Relish	CCTGTGAAGACATTAGGAGGAGTA	CCAGTTGTGGCATTCTTTAGG
Imd	TCACATTGGCCCCGTTATCC	ATCTCGCGACTGCACTTCAA
HSP70	AACGATTCTCAGCGTCAGG	AGGTGCCACGGAACAGAT
TGF-β	AACCATGCCCTTGTGCAAAC	CTTTGGGGGAACCTCGGTC
IL-1β	TGTGACCACCATCCACCAGAAC	GATCCCGCAGTAACCGAATAAG
TNF	AAAGAGGAACGTGGTCATGG	CACTCCTTTCCCCACTGTGT
IFN-γ	GACTTCGGTGCCACGGAACAAG	GACGCTCACTTTCACGCGGTCT
AIF	GCTGACATCATCCCCAACT	CTGGAATGTGCTATGGTG

LZM, lysozyme; LGBP, lipopolysaccharide and β-1,3-glucan-binding protein; HSP70, heat shock protein 70; IMD, innate immune deficiency; TGF-β, transforming growth factor-β; IL-1β, Interleukin-1β; TNF, tumor necrosis factor; IFN-γ, interferon-γ; AIF, apoptosis inducing factor.

### 2.4 Immune and antioxidant capacity analysis

The intestine and serum samples were used for measurement of the activities of acid phosphatase (ACP), alkaline phosphatase (AKP), lysozyme (LMZ), total superoxide dismutase (SOD), catalase (CAT), and glutathione peroxidase (GPx), and the contents of reduced glutathione (GSH) and malondialdehyde (MDA) using commercial diagnostic kits (Nanjing Jiancheng Bioengineering Institute, China). The phenoloxidase (PO) parameter in the intestine and serum was assayed by a competitive ELISA kit produced by Nanjing Camilo.

### 2.5 Statistical analysis

The growth performance of shrimp was determined by final weight, Length gain rate (%), Specific growth rate (%/d) and Condition factor (g/cm^3^). The formulas were defined as follows:


; Length gain rate (%) =100 × (final length – initial length)/initial size

; Specific growth rate (%/d) =100 × (ln final weight − ln initial weight)/days

Condition factor (g/cm^3^) = final weight/final length^3^.

The data for gene expression, immune and antioxidant parameters, and growth were analyzed by student’s t-test using SPSS version 26 software. Data are presented as mean ± standard error of the mean (SE). Statistical significance was determined at *P< 0.05*.

## 3 Results

### 3.1 The intestinal microbiota of white shrimp

#### 3.1.1 The composition of the intestinal microbial community in white shrimp

**Figure 1:**
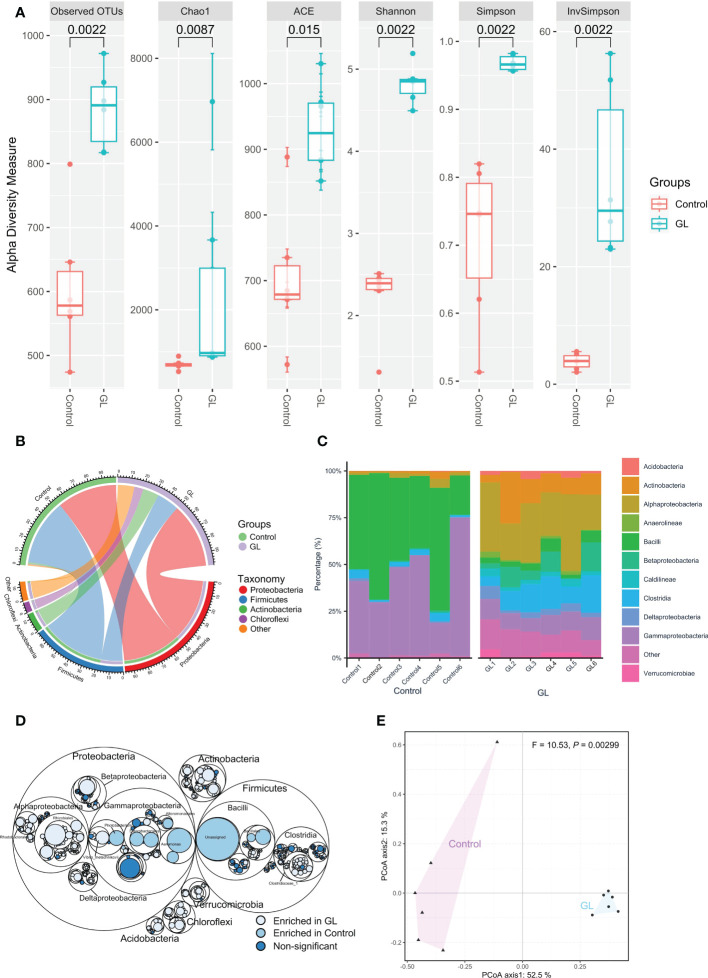
The effects of β-1,3-glucan on the composition of intestinal microbial community of *Litopenaeus vannamei*. **(A)** The α-diversity of the intestinal microbiota; **(B)** The relative abundance of species above ≥ a cutoff value of 2%; **(C)**The close lots of the top 10 classes; **(D)** Maptree plot descriptions of the taxonomic differences. The giant circles represent species level; the inner circles represent class, order, family, and genus for the panel; **(E)** PCoA analysis visualizing dissimilarities in the intestinal microbial community through permutational analysis of variance (PERMANOVA) based on the Bray–Curtis distance.

Compared to the Control group, the shrimp in the GL group exhibited a significantly higher microbial α-diversity in terms of a higher value in Observed OTUs, Chao1, AEC, Shannon, Simpson, and in InvSimpson (*P< 0.05*, **Figure 1A**). As shown in **Figure 1B**, Proteobacteria (Control: 46.43, GL: 50.12%), Firmicutes (Control: 50.80%, GL: 17.47%), and Actinobacteria (Control: 1.35%, GL: 12.47%) took dominate in the intestinal microbial community. Mainly, Gammaproteobacteria (43.25%) and Bacilli (47.80%) were observed as the predominant classes in the shrimp-fed basal diet. In contrast, shrimp in the GL group was predominated by Alphaproteobacteria (28.58%), Clostridia (12.81%), Actinobacteria (12.46%), Gammaproteobacteria (9.96%), and Betaproteobacteria (7.58%, **Figure 1C**). Shrimp fed basal diet possessed a significantly higher proportion of Photobacterium, Enterobacteriales and Aeromonadales from Gammaproteobacteria, and Bacillales and Unassigned microbes from Bacilli (*P< 0.05*). In contrast, Rhodobacterales and Rhizobiales from Alphaproteobacteria and Clostridiaceae_1 from Clostridia were detected dramatically higher in the GL group, with a notably higher percentage of microbes from Acidobacteria, Verrucomicrobia, Chloroflexi, and Actinobacteria (*P< 0.05*, **Figure 1D**). Meanwhile, the PCoA analysis further confirmed the significant separation in the microbial community between Control and GL groups (*P* = 0.00299, **Figure 1E**).

#### 3.1.2 The microbial interactions within the intestinal microbial community of white shrimp

**Figure 2:**
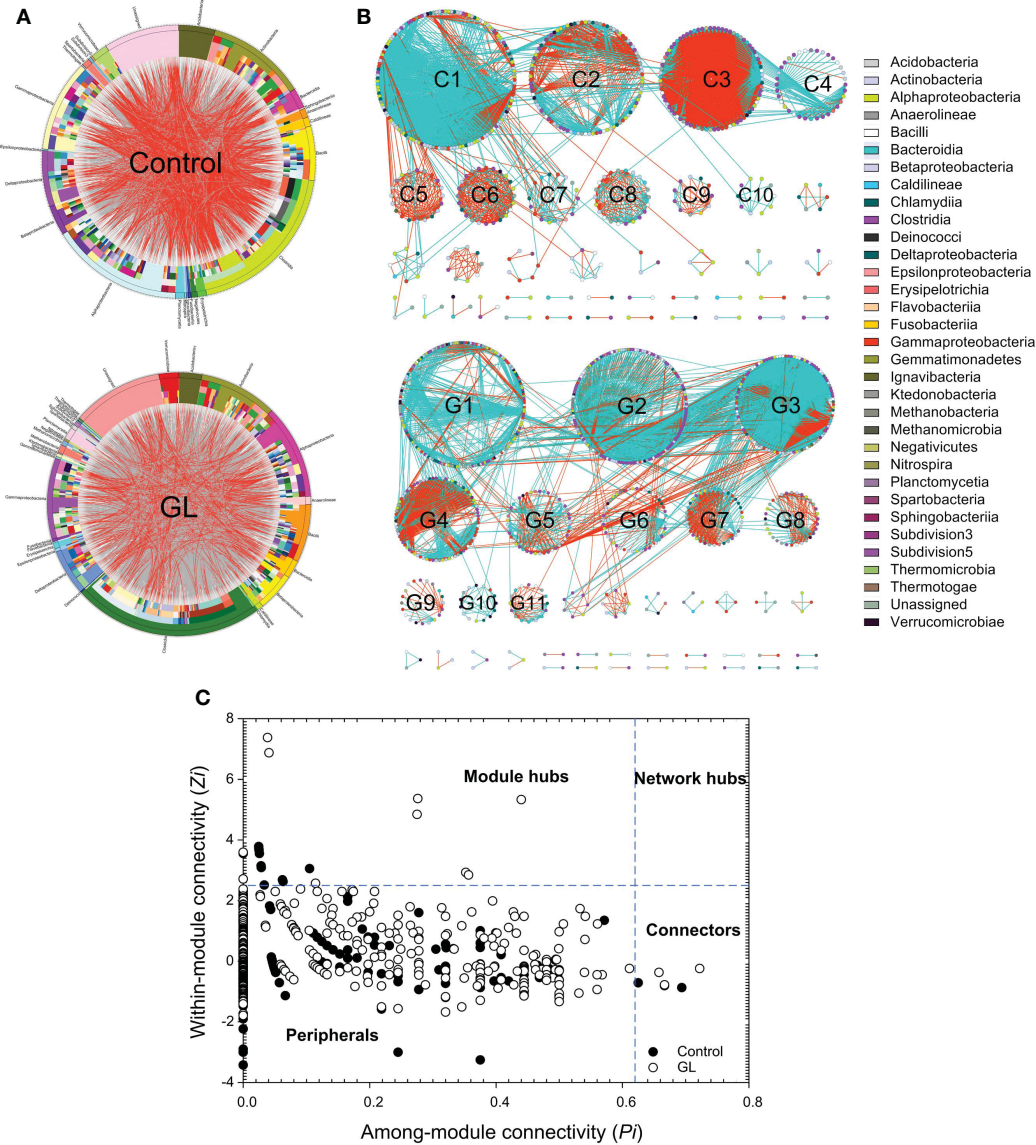
The effects of β-1,3-glucan on the interspecific interactions within the intestinal microbial community of *Litopenaeus vannamei*. **(A)** Circular plot descriptions of the interaction between different species. The taxonomic levels were class, order, family, genera, and species from the outside to the inside of the circle; **(B)** Ecological network within the microbial community. The edges (gray edge = positive interaction and red edge = negative interaction) inside the circle and ecological network represent the interactions between species. **(C)** Z-P plot showing the distribution of OTUs based on their topological roles.

Except for the notable variation in the microbial community, the circus plot exhibited the species-species interactions (red edges: negative interactions, gray edges: positive interactions) across different OTUs (Control: 558 OTUs, GL: 813 OTUs) from 32 bacterial classes within the intestinal microbial community (**Figure 2A**). The predominate classes, including Clostridia, Alphaproteobacteria, Actinobacteria, Gammaproteobacteria, and Bacilli, were observed to dominate the ecological networks, in which 10 and 11 modules (≥ 10 OTUs) were respectively attended in Control and GL networks as shown in **Figure 2B**. GL network was dominated by positive interactions, whereas opposite results were followed in the Control network with submodules C2, C3, C5, C6, C8, and C9 predominated by negative interactions. Within the network, the Control group has 10 module hubs and 3 connectors, while 11 module hubs and 3 connectors were found in the GL group (**Figure 2C** and **Table 3**).

**Table 3:** The composition of the ecological network.

Index	Control	GL
Acidobacteria	24	23
Actinobacteria	66	78
Alphaproteobacteria	88	95
Anaerolineae	6	8
Bacilli	36	58
Bacteroidia	13	18
Betaproteobacteria	20	38
Caldilineae	6	6
Chlamydiia	0	1
Clostridia	109	191
Deinococci	0	1
Deltaproteobacteria	37	46
Epsilonproteobacteria	1	3
Erysipelotrichia	6	6
Flavobacteriia	0	1
Fusobacteriia	2	3
Gammaproteobacteria	61	81
Gemmatimonadetes	0	2
Ignavibacteria	1	3
Ktedonobacteria	0	1
Methanobacteria	0	8
Methanomicrobia	0	5
Negativicutes	4	1
Nitrospira	1	1
Planctomycetia	6	12
Spartobacteria	5	4
Sphingobacteriia	1	4
Subdivision3	2	1
Subdivision5	1	1
Thermomicrobia	0	1
Thermotogae	1	1
Verrucomicrobiae	11	20
Unassigned	50	91
Total number of OTUs	558	813
The number of modules (≥10 OTUs)	10	11
The number of connectors	3	3
The number of gray edges	1880	3524
The number of red edges	1967	1219
Total number of edges	3847	4743

#### 3.1.3 The metabolic function of intestinal microbial community of white shrimp

**Figure 3:**
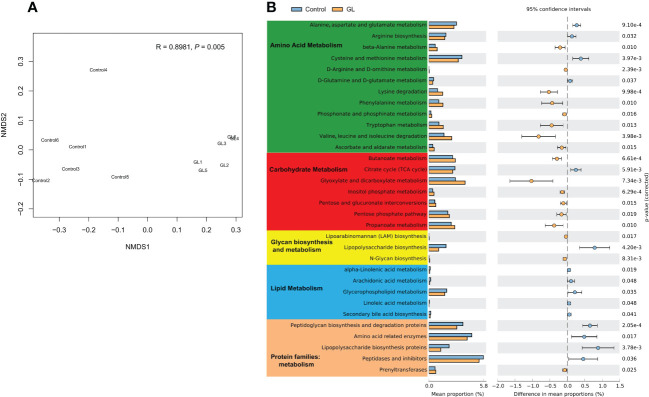
The effects of β-1,3-glucan on the microbial function. **(A)** NMDS plot visualizing microbial function dissimilarities using Bray–Curtis distance at KO level, and the test of difference was calculated by analysis of similarity (ANOSIM); **(B)** A two-sided Welch’s t test analysis of the microbial metabolic function using the response ratio method at a 95% confidence interval (CI).

The microbial function in the GL group dramatically differed from that in the Control group at KO levels (P = 0.005, **Figure 3A**). To explore the response of microbial metabolism to the diets, the KEGG functional categories were further analyzed, mainly focusing on amino acid metabolism, carbohydrate metabolism, lipid metabolism, and protein families: metabolism (**Figure 3B**). Compared to the shrimp-fed basal diet, much more metabolic pathways related to the amino acid and carbohydrate metabolisms of intestinal microbiota were notably increased in the shrimp-fed β-1,3-glucan supplementation diet (*P< 0.05*). The microbial lipopolysaccharide biosynthesis and lipid metabolism were more active in the Control group together with more proteins related to metabolism (*P< 0.05*), especially the lipopolysaccharide biosynthesis proteins.

### 3.2 Gene expression related to inflammation and immune in the intestinal tissue of white shrimp

**Figure 4:**
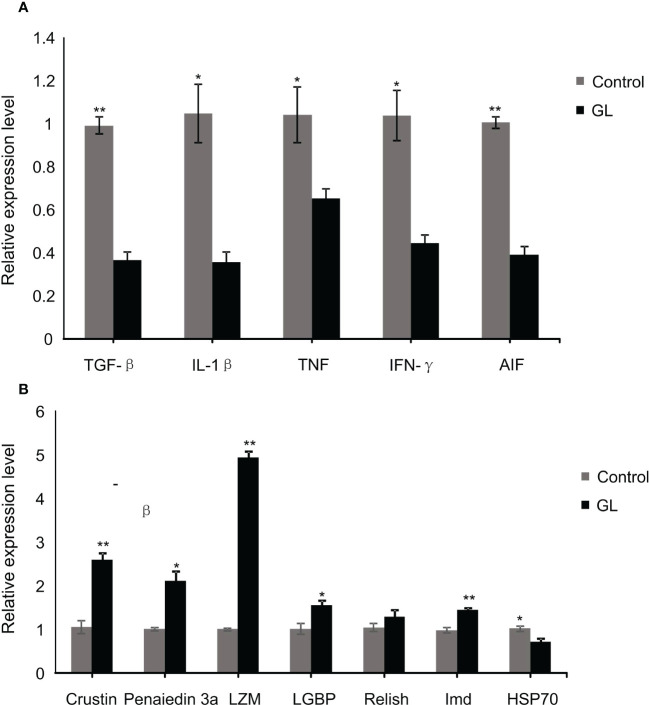
The relative mRNA levels of immune **(A)** and inflammation **(B)** related genes in intestinal tissue of *Litopenaeus vannamei*. *P< *0.05*, **P< *0.01*.

Compared to the shrimp fed basal diet, a significant reduction in the mRNA transcription level of inflammatory factor genes, including TGF-β, IL-1β, TNF, IFN-γ, and AIF, was detected in the intestinal tissue of shrimp in glucan diet (*P< 0.05*, **Figure 4A**). The β-1,3-glucan significantly induced the overexpression of immunity genes, including Cristin, Penaiedin 3a, LMZ, LGBP, and Imd in the intestinal tissue of shrimp (*P< 0.05*), while it unaffected the gene expression of Relish (**Figure 4A**); meanwhile, the expression of heat shock protein gene HSP70 in the intestinal tissue was dramatically suppressed in GL group (*P< 0.05*).

### 3.3 The immunity, antioxidant capacity, and growth performance of white shrimp

The β-1,3-glucan supplementation enhanced the concentration of PO and the activities of AKP, ACP, and LMZ in intestinal tissue. Significant differences in the value of activities of AKP, ACP, and LMZ were found between GL and Control groups (*P*< *0.05*, **Table 4**). Similarly, significantly higher activities of serum AKP, ACP, and PO have been detected in shrimp from glucan group (*P*< *0.05*). Compared to the Control group, the actions of CAT, TSOD, and GPx were notably elevated in the intestine tissue of shrimp from the GL group together with a reduction in MDA content (*P< 0.05*, **Table 5**); meanwhile, the GL group recorded higher activities of serum CAT and GPx, followed by a notably lower MDA content. Additionally, compared to the Control group, shrimp fed β-1,3-glucan diet exhibited significantly higher values in Final weight, Final length, Length gain, and Specific growth rate (*P*< *0.05*), concurrent with a notable reduction in Condition factor (*P*< *0.05*, **Table 6**).

**Table 4:** The effects of β-1,3-glucan on the immunity of *Litopenaeus vannamei*.

Parameters	Control	GL	P value
Intestine			
AKP (King unit/gprot)	50.12 ± 4.46	100.82 ± 10.62	0.006
ACP (King unit/gprot)	160.05 ± 8.19	204.13 ± 14.5	0.021
LZM (U/mgprot)	29.93 ± 1.9	37.87 ± 1.46	0.011
PO (ng/gport)	22.3 ± 3.15	46.89 ± 11.65	0.09
Serum			
AKP (King unit/100ml)	5.41 ± 0.62	12.29 ± 1.7	0.004
ACP (King unit/100ml)	3.40 ± 0.3	9.91 ± 2.23	0.033
LZM (U/ml)	25.00 ± 4.3	36.11 ± 6.33	0.177
PO (U/L)	85.99 ± 4.67	132.78 ± 6.55	<0.001

**Table 5:** The effects of β-1,3-glucan on the antioxidant capacity of *Litopenaeus vannamei*.

Parameters	Control	GL	P value
Intestine			
CAT (U/mgprot)	6.85 ± 0.91	10.39 ± 1.58	0.089
TSOD (U/mgprot)	24.92 ± 2.56	33.98 ± 2.32	0.025
GPx (U/mgprot)	15.74 ± 1.59	39.43 ± 3.18	<0.001
GSH (mg/gprot)	11.93 ± 1.52	15.06 ± 1.25	0.143
MDA (nmol/gport)	1861.11 ± 202.84	959.56 ± 73.84	0.005
Serum			
CAT (U/mL)	23.84 ± 2.31	31.67 ± 1.43	0.015
TSOD (U/mL)	704.23 ± 16.51	751.51 ± 14.21	0.055
GPx (U/mL)	1109.38 ± 48.99	1296.88 ± 19.62	0.005
GSH (mg/mL)	26.14 ± 3.95	59.24 ± 6.45	0.005
MDA (nmol/ml)	22.37 ± 1.4	15.79 ± 1.92	0.02

**Table 6:** The effects of β-1,3-glucan on the growth performance of *Litopenaeus vannamei*.

Parameters	Control	GL	P value
Initial weight (g)	1.19 ± 0.04	1.23 ± 0.01	0.402
Final weight (g)	10.62 ± 0.36	12.21 ± 0.22	<0.001
Final length (cm)	11.7 ± 0.1	12.53 ± 0.08	<0.001
Length gain rate (%)	1069.52 ± 10.34	1153.04 ± 8.35	<0.001
Specific growth rate (%/d)	8.74 ± 0.04	8.93 ± 0.02	0.001
Condition factor (g/cm^3^)	0.66 ± 0.01	0.62 ± 0.01	0.007

## 4 Discussion

Multitudinous pieces of evidences from the animal model and human studies have verified the critical role played by intestinal microbiota in intestinal health, and the diet consumed by the host could shape the microbial composition (25, 28, 35, 36). β-glucan serves as a prebiotic and is confirmed to positively impact the intestinal microbiota and the host’s health (19). Here, β-1,3-glucan supplementation did affect the intestinal microbiota and significantly increased the microbial diversity in white shrimp. Similar results were also observed in tilapia (21), carp (*Cyprinus carpio*) (37) and white shrimp from a recent study (22). Although the composition of the intestinal microbial community can be dramatically affected by the diets, the core microbiota is essentially stable in the host (38). Here, results indicated that white shrimp fed basal and β-1,3-glucan diet also shared the same core microbiota from Proteobacteria and Firmicutes, even though intestinal microbiota structure was notably altered by dietary supplementation of β-1,3-glucan. The current results suggested that shrimp fed with commercial feed displayed a significant increase in the percentage of Aeromonas species from Gammaproteobacteria, which were confirmed to be opportunistic pathogens in freshwater-farmed white shrimp (2); β-1,3-glucan diet was found to inhibit these Aeromonas species significantly, and dramatically increase the proportion of microbes from Alphaproteobacteria, Betaproteobacteria, Deltaproteobacteria, Clostridiaceae_1, and scarce class groups like Actinobacteria, Chloroflexi, Verrucomicrobia, and Acidobacteria. The increased ratio of these low-class groups led to the rise of the microbial diversity in shrimp.

The interspecific interactions capacitate the microbial community to form an ecological network through which the micro-ecological ecosystem maintains its dynamic balance in intestine (23). In the present study, shrimp fed basal and β-1,3-glucan diets displayed two completely different patterns of microbial interactions referred to as competition and cooperation dominated in submodules of the ecological network, respectively. Based on the r/K selection theory in ecology, the r-strategy species would be the representative community appearing in nutrient-rich environments with characteristics of low competition, high nutrient utilization capacity, and high growth rate (39). Although artificial feeding provided a nutrient-rich environment for the intestinal microbiota, the proliferation of Aeromonas species, the pathogens, intensified the competition within the microbial community for the nutrients in this study. In spatial conditions, the cooperation-dominated communities are more stable because the cooperative interactions are more robust to population disturbance, while the competitive interactions are susceptible to perturbations (40, 41). And thus, the β-1,3-glucan diet promoted intestinal microbiota homeostasis by inhibiting the competition within the microbial community through the suppression of Aeromonas. The dominant microbiota is the main component of the ecological network, in which the generalists referred to connectors and module hubs serve as structural and functional keystones and execute a key role in maintaining the property of the web (42). Therefore, β-1,3-glucan promoted the stability of ecological network by increasing the number of module hubs. Taken together, dietary supplementation of β-1,3-glucan could improve the homeostasis of intestinal microbiota in white shrimp through the inhibition of composition caused by Aeromonas and the increase in number of module hubs.

The intestinal microbiota plays a vital role in host nutrient metabolism since microbial fermentation and nutrient synthesis provide numerous metabolites (25–27). Undoubtedly, the variation in microbial composition would further cause a change in microbial metabolism. The microbial carbohydrate metabolism was more active in the white shrimp fed a β-1,3-glucan diet in response to polysaccharide supplementation. Meanwhile, due to the significant reduction in the ratio of Aeromonas, the microbial lipopolysaccharide biosynthesis was significantly inhibited in shrimp fed β-1,3-glucan diet in this study. As we know, lipopolysaccharide could induce intestinal inflammation by triggering TLR4-mediated inflammatory pathway (43, 44), and this may explain why β-1,3-glucan diet displayed an anti-inflammatory capacity in white shrimp in terms of the significant decrease in expression of inflammatory factor genes in this study. Additionally, β-1,3-glucan also could suppress intestinal inflammation by elevating the activity of intestinal AKP in the present study since the AKP can remove the phosphoric acid groups from lipopolysaccharide, thereby reducing its inflammatory effects (45, 46). Therefore, dietary supplementation of β-1,3-glucan could benefit intestinal health by recovering intestinal dysfunction induced by an inflammatory response.

The β-glucan, as an ideal immunostimulant, is widely applicated in aquaculture and positively impacts the immunity and antioxidant system of aquatic animals. Pieces of evidence from previous studies in white shrimp have confirmed the immune-enhancing effect of β-1,3-glucan (8, 47). The lipopolysaccharide and β-1,3-glucan binding protein (LGBP), vital pattern recognition proteins, can recognize lipopolysaccharide and β-1,3-glucan and subsequently trigger innate immunity (48). Indeed, the present results suggested that the expression of intestinal LGBP was significantly increased in shrimp fed β-1,3-glucan diet, together with an enhancement in immunity parameters in intestinal tissue and serum. Additionally, β-1,3-glucan supplementation enhanced the antioxidant capacity of shrimp, and similar results were also observed in white shrimp fed β-1,3-glucan (7). HSP70 serves as an important oxidative stress biomarker (49), and higher expression of HSP70 in the intestinal tissue shrimp fed β-1,3-glucan confirmed the antioxidant property of β-glucan again. Along with the improvement of intestinal function, the growth performance of white shrimp was also dramatically promoted by β-1,3-glucan finally.

In conclusion, β-1,3-glucan supplementation significantly altered the microbial composition and promoted the homeostasis of intestinal microbiota, and inhibited intestinal inflammatory through the suppression of pathogenic bacteria Aeromonas, concurrent with an enhancement in the immunity, antioxidant capacity, and growth performance of white shrimp.

## Data availability statement

The datasets presented in this study can be found in online repositories. The names of the repository/repositories and accession number(s) can be found below: https://www.ncbi.nlm.nih.gov/, PRJNA908810.

## Ethics statement

The animal study was reviewed and approved by Nanchang University.

## Author contributions

GY and MP designed the experiments and supervised the manuscript. KS and LB carried out the animal experiment and sample analysis with the help of ML, WL, QZ, JD, PF, BH, CW, and wrote the manuscript. VK revised the manuscript. All authors read and approved the final manuscript. All authors contributed to the article and approved the submitted version.
